# Catalytic transformation of ethanol into 1,3-butadiene

**DOI:** 10.1186/s13065-014-0053-4

**Published:** 2014-09-10

**Authors:** Matthew D Jones

**Affiliations:** Department of Chemistry, University of Bath, Claverton Down, Bath, BA2 7AY UK

**Keywords:** Ethanol, 1,3-butadiene, Heterogeneous, Catalysis

## Abstract

1,3-Butadiene is an important constituent of many products that we rely upon. It is currently prepared from non-sustainably derived sources. However, in the early part of the 20^th^ Century the use of ethanol as a source of 1,3-butadiene has been reported. With the arrival of a cheap and bountiful supply of crude-oil derived sources the need for the sustainable route was deemed unnecessary. In recent years the conversion of ethanol to 1,3-butadiene has undergone somewhat of a mini resurgence as the chemical industry looks to try to find a sustainable and secure route to this important building block. This review will emphasise some of the most recent work in the field and look ahead to what needs to be achieved to make this research a reality.

**Graphical Abstract:**
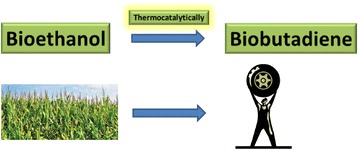
This review focusses on the recent progress in the area of the conversion of ethanol to 1, 3-butadiene.

This review focusses on the recent progress in the area of the conversion of ethanol to 1, 3-butadiene.

## Introduction

**Scheme 1:**
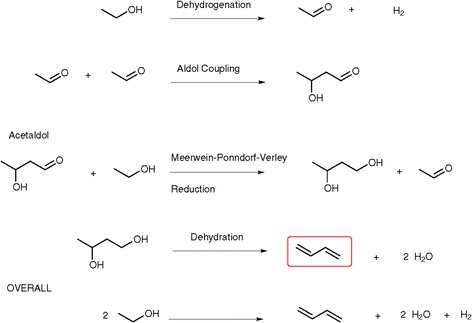
**Steps involved in the conversion of ethanol to 1,3-BD.**

**Scheme 2:**
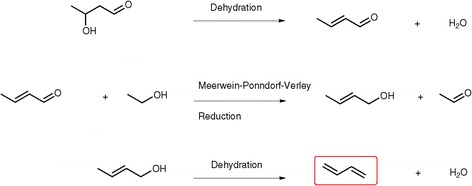
**Steps in involved in the conversion of ethanol to 1,3-BD.**

1,3-Butadiene (1,3-BD) is an important building block in many chemical processes. Its main use is as a monomer for the production of synthetic rubbers [1]. The most significant use of 1,3-BD is in the manufacturing of styrene-butadiene (SBR) rubbers which are primarily used in the production of tyres. Currently, 1,3-BD is mainly formed as a by-product of the naphtha steam cracking process – a co-product of ethene manufacturing – with 1,3-BD being isolated after costly extractive distillation steps. 1,3-BD can also be produced by the dehydrogenation of butane or butene (Houdry process) [1,2]. In recent years the cost of 1,3-BD has fluctuated massively and the price has increased, for example the cost of a tonne of 1,3-BD was ca. $1,500 USD in March 2014 compared to $850 USD in August 2013. The cost (and fluctuation) is not sustainable in the long term for the major users of this important building block. The increase in cost can be attributed to several factors i) the increasing rise in the price of crude oil; ii) the move to lighter feedstocks from the cracking process and iii) the “shale-gas” surge in Europe and the US [3]. Shale gas contains ethane which can be dehydrogenated to ethene – which is a 1,3-BD free route and consequently causing a reduction in ethene production from the steam cracking route and, hence, 1,3-BD. This necessitates the need to produce 1,3-BD *via* a bio-based route. Whilst “shale-gas” has its sceptics it may well necessitate the increased development of 1,3-BD from renewable sources. Increased dependence on “shale-gas” as a source of natural gas may lead to acute shortfalls in the supply of key starting materials for industry that are currently sourced from more traditional feedstocks. Paradoxically, the chemical industry needs to look back in time to find a solution to this problem. The former USSR and the US developed a one-step and two-step process for the conversion of ethanol into 1,3-BD, Scheme 1:

The mechanism is still open to much debate, but it is generally accepted that the following are key to its production. Initially, ethanol is dehydrogenated to afford acetaldehyde then an aldol process can occur between two acetaldehydes to form acetaldol. A Meerwein-Ponndorf-Verley (MPV) reduction step can then follow (also generating more acetaldehyde that can be used in the aldol step) to form 1,3-butandiol, subsequent dehydration forms butadiene. It is obviously possible that if the final two steps are reversed (Scheme 2), and acetaldol is dehydrated to form crotonaldehyde this can than undergo a MPV reduction to form crotylalcohol, which can be dehydrated giving 1,3-BD. Clearly, there are many undesired processes that can be occurring alongside the desired path forming a plethora of side products. By-products of this process include (but are not limited to) – acetone, propane/propene, butenes, diethyl ether, pentenes, hexenes, ethyl acetate, butanol and ethane/ethene. Acetaldehyde can also be thought of as a by-product, but this can be recycled into the feed and re-used. The financial viability of the process will depend upon minimising the formation of by-products and thus maximising the overall process yield of 1,3-BD.

The success of this technology will depend on a cheap and readily available source of ethanol [4]. As with all processes relying on sustainable and renewable resources the new route must not impart undesired consequences on existing supply chains or impact on food provisions and security. With the arrival of algal biomass for bioethanol production then the possible environmental concerns can be circumvented [5]. The sustainable assessment (techno-economic) of the ethanol to 1,3-BD process has also been investigated by Patel and co-workers, who found that the bio-based route compares favourably with the traditional naphtha-based route [6,7].

## Review

The conversion of ethanol to 1,3-BD is not a new process – with much work being carried out in the early part of the 20^th^ Century. However, with the advent of a cheap and seemingly plentiful supply of crude-oil this research fell out of favour. In the 1980s it became more popular and in the 21^st^ Century it is essential that we fully make use of this chemical technology. Thus, this short review will highlight the most recent examples of catalytic investigations into this process, the seminal initial work of Lebedev, Corson, Bhattacharyya and Ostromislenskiy has been the subject of review articles [8,9] and the reader is directed towards those and the primary literature for further details [10-21]. However, the scope of this review is to look at the most current literature in the area – the review focusses on sepiolite, MgO-SiO_2_ and pure SiO_2_ supported catalysts.

### Post 1980’s Catalytic conversion of Ethanol to 1,3-BD

**Scheme 3:**
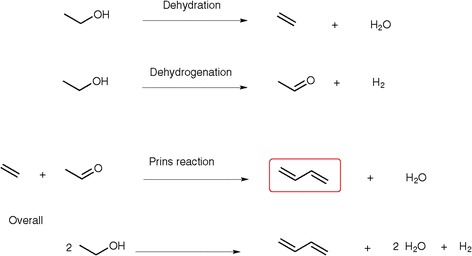
**Mechanism for the production of 1,3-BD from ethanol proposed by Fripiat.**

In 1981 Kitayama and co-workers investigated the catalytic activity of sepiolite {(H_2_O)_4_(OH)_4_Mg_8_Si_12_O_30_.6-8H_2_O} for the production of 1,3-BD [22]. Sepiolite was chosen as it is relatively easy to exchange the Mg(II) centres in the material with transition metals. In their study Mn(II) exchanged materials were investigated. The most promising results were observed at a temperature of 300°C with a 33.4% selectivity towards 1,3-BD, with 41.4% ethene. This is comparison to just 2.4% 1,3-BD selectivity for the pure sepiolite material. The high quantity of ethene is a problem for this catalyst system and ethene should be avoided. Fripiat further investigated the substitution of sepiolite with either vanadyl or silver cations [23,24]. In the vanadyl case they observed only a modest selectivity to 1,3-BD when pure ethanol was used as the feed [23]. However, when the feed was rich in acetaldehyde a high selectivity, ca. 80%, to 1,3-BD was achieved. This was observed with and without the presence of the vandyl cation on the aluminated sepiolite. They proposed that the mechanism for this process involves the Prins reaction, Scheme 3:

For the silver aluminated sepiolite they observed that both the ethene and 1,3-BD selectivities increase linearly with total conversion [24]. They attributed this observation as support for the Prins mechanism. The Prins mechanism is an attractive approach as this can utilise the ethene by-product. However, since these initial studies the role of the Prins reaction has not been further investigated. The use of zeolites for this process is also not widely employed, this is presumably related to the high quantity of low-value ethene produced due to the acidic properties of most zeolites.

Magnesia-silica materials have been shown to be very effective catalysts (either pure supports or with added metal centres) for this process [25-27]. The magnesia offers basic sites and the silica acidic sites. It is hypothesised that the magnesia enhances the aldol and dehydrogenation steps of the mechanism, whereas the silica assists the dehydration steps on the mechanism. The preparation method and molar ratio of MgO-to-SiO_2_ have proved to be of critical importance in this field. For example, Ohnishi prepared MgO:SiO_2_ (with a 1:1 molar ratio) in three difference ways and observed yields of 1,3-BD ranging from 2–42% (T = 350°C, Flow EtOH = 6.5 × 10^−4^ mol h^−1^) [27]. It was observed that the most effective catalyst was prepared by the wet kneading of Mg(OH)_2_ {prepared by hydrolysis of Mg(NO_3_)_2_ with NH_3_} with SiO_2_ {prepared from hydrolysis of Si(OEt)_4_ with HNO_3_, EtOH and NH_3_}. Using MgCl_2_ as the source of MgO resulted in significantly poorer yields of 1,3-BD. Interestingly, with the addition of 0.1 wt% of either Na_2_O or K_2_O then the selectivity to 1,3-BD significantly increases. This maybe due to the reduction in the Brϕnsted acidity of the support, highly Brϕnsted acidic materials are well known to catalyse the conversion of EtOH to ethene and diethyl ether. MgO:SiO_2_ catalysts were also studied by Kvisle et al., the preparation of which was analogous to that of Ohnishi. Their catalytic tested were performed at 350°C, flow EtOH = 8 μl h^−1^, mass of catalyst 200 mg [26]. They observed that as the flow of EtOH increases then the conversion decreases and the selectivity to 1,3-BD also decreases. The selectivity to 1,3-BD could be increased by the addition of acetaldehyde to the feed or, interestingly, adding oxygen to the carrier gas. The addition of a second alcohol (methanol or isopropanol) did not alter the yield of 1,3-BD. It was thus hypothesised that the rate limiting step is not the hydrogen transfer step, which would be enhanced with the addition of other alcohols. Thus, the rate determining step occurs prior to the MPV step. It was also noted that the wet-kneaded catalyst significantly out performs (in terms of selectivity and longevity) a mechanical mixture of MgO and SiO_2_ illustrating that there is a synergistic effect between the two oxides. Kitayama also prepared large surface area (up to 784 m^2^g^−1^) nickel magnesium silicate materials for this process [28]. The catalysts were prepared with a 10 wt% NiO content, it was found that the optimum ratio of Si/Mg was 1.5, which afforded a 31% yield to 1,3-BD and a very low selectivity to ethene of 0.5%. It is believed that this ratio gives the optimum ratio of acid-to-base sites to enhance the selectivity to 1,3-BD. This was attributed to the decrease in the acid sites. Recently, Sels added transition metal centres to MgO:SiO_2_ materials [25]. In their study the optimal ratio of Mg/Si was 2. Adding CuO, ZnO and Ag to this support (T = 350°C, EtOH concentration = 1.5 × 10^4^ppm) was seen to have a positive effect on selectivity. The preparation of these ternary systems was also investigated and it was found that the MgO:SiO_2_ must be prepared first followed by addition of the transition metal. Recently, Jones and co-workers have prepared a series of bimetallic (ZnO/ZrO_2_) supported MgO:SiO_2_ catalysts [29]. For the undoped materials an optimum ratio of Mg:Si was 2:1 (T = 325°C, WHSV = 0.3 g_EtOH_/g_cat_h, mass of catalyst = 1.0 g). However, this was not the case for the case for the bimetallic catalyst and the optimum ratio was 95:5 respectively. Interestingly, a small amount of SiO_2_ is essential for this process, with 100% MgO the conversion was 5% with a 19% selectivity to 1,3-BD compared to 30% and 68% for the 95:5 material. There are earlier examples of the exploitation of MgO-SiO_2_ by Niiyama who showed that an 85:15 ratio was optimal for the process [25]. They observed that the rate determining step was acetaldehyde formation which was catalysed by the basic sites in the material. They argue that it is important to control the acidity and basicity of the catalyst. [30] Takezawa have also studied the mechanism for the conversion of ethanol to acetaldehyde on MgO supports, and the formation of an ethoxide species on the MgO was observed *via* IR spectroscopy [31].

Supports such as SiO_2_ and Al_2_O_3_ have also been utilised in the last three years for this process [32-35]. Jones has developed a series of bi- and tri-metallic catalysts for this reaction. They observed that with EtOH as the feed (LHSV = 1.0 h^−1^, T = 375°C) a ZrO_2_:ZnO:CuO:SiO_2_ (1 wt% of each metal) catalyst was the most efficient giving a 67.4% selectivity to 1,3-BD [34]. Interestingly, as the pore diameter (40 – 60 – 150 Å) of the porous SiO_2_ increased then the selectivity to 1,3-BD increased. This was attributed to the acidity of the support decreasing, thus reducing the quantity of ethene formed. It was hypothesised that the ZnO was active for the dehydrogenation and the ZrO_2_ the aldol coupling step. Ordomskiy and co-workers have patented a series of gold, silver or copper and a metal oxide from magnesium, titanium, zirconium or tantalum deposited on SiO_2_ [35]. They achieved upto a 82% yield of 1,3-BD (T = 325°C, WHSV = 0.3 g_EtOH_/g_cat_h, 1:10 acetaldehyde to ethanol) with a Au-ZrO_2_-SiO_2_ material. They also observed high yields with Ag and CeO_2_ containing catalysts. Carbon (coking) formation is also possible for this process and will block access to active sites and thus reduce activity. This will require regeneration of the catalyst and periodic production downtime. Ezinkwo has shown that if H_2_O_2_ is added as a “process initiator” then this is a potential solution to coking issues [33]. Without the addition of the process initiator the catalyst was only lasted for 48 hours on stream. However, with the addition of H_2_O_2_ the catalyst activity persisted for 120 hours without reduction, indicating the possibility of running this catalysis in a continuous operation. The catalyst used in this study was a ZnO/γ-Al_2_O_3_ system. In 2014, inspired by work in the early 20^th^ Century, Chae and co-workers produced Ta_2_O_3_- supported on ordered mesoporous silica (SBA-15, MMS and KIT-6) [32]. They achieved conversions upto 47% and selectivity to 1,3-BD of 80% (T = 350°C, LHSV = 1 h^−1^). Importantly, it was observed that catalysts based on these ordered materials showed higher tolerances towards coke and higher activity.

## Conclusions

The conversion of ethanol to 1,3-butadiene is becoming increasingly important in the 21^st^ Century. However, for the opportunities that this process offers to be fully realised the following problems still remain:

1. Increasing the selectivity, one of the most significant costs associated with the industrial scale-up of this process will be the separation of the 1,-BD from the by-products. Thus, the higher the selectivity then potentially the lower the cost of separation. The current literature clearly demonstrates that there needs to be the “goldilocks” condition of “just the right” balance of acid and base sites in the catalyst. It is anticipated that selectivities in excess of 70% will be required. This is a very challenging target given the plethora of possible side reactions.
2. The mechanism has been much debated in the literature. Future studies are required to fully ascertain the respective rates of the Prins mechanism compared to the aldol/hydrogen transfer process. These could entail isotopic labelling studies and/or computational analysis to fully probe which steps of the mechanism are occurring on which active site of the catalyst.
3. Reactor engineering, once the optimum catalyst has been developed studies are urgently required to develop reactors for this process – the relative merits of a fixed bed or fluidised bed reacts still needs to be investigated further [10]. Furthermore, work needs to be directed towards complications with coking [33].

## References

[1] White WC (2007). Butadiene production process overview. Chem Biol Interact.

[2] Mascal M (2012). Chemicals from biobutanol: technologies and markets. Biofuels Bioprod Biorefining.

[3] Bruijnincx PCA, Weckhuysen BM (2013). Shale gas revolution: an opportunity for the production of biobased chemicals?. Angew Chem-Int Engl.

[4] McMillan JD (1997). Bioethanol production: Status and prospects. Renew Energ.

[5] John RP, Anisha GS, Nampoothiri KM, Pandey A (2011). Micro and macroalgal biomass: A renewable source for bioethanol. Bioresour Technol.

[6] Patel AD, Meesters K, Den Uil H, De Jong E, Blok K, Patel MK (2012). Sustainability assessment of novel chemical processes at early stage: application to biobased processes. Energy Environ Sci.

[7] Patel AD, Meesters K, Den Uil H, De Jong E, Worrell E, Patel MK (2013). Early-stage comparative sustainability assessment of new bio-based processes. ChemSusChem.

[8] Angelici C, Weckhuysen BM, Bruijnincx PCA (2013). Chemocatalytic conversion of ethanol into butadiene and other bulk chemicals. ChemSusChem.

[9] Kozlowski JT, Davis RJ (2013). Heterogeneous catalysts for the guerbet coupling of alcohols. ACS Catal.

[10] Bhattacharyya SK, Avasthi BN (1966). Catalytic conversion of ethanol to butadiene by two-step process in fluidised bed. J Appl Chem.

[11] Bhattacharyya SK, Sanyal SK (1967). Kinetic study on mechanism of catalytic conversion of ethanol to butadiene. J Catal.

[12] Bhattacharyya SK, Avasthi BN (1963). 1-step catalytic conversion of ethanol to butadiene in a fluidised bed. Ind Eng Chem Process Des Dev.

[13] Corson BB, Jones HE, Welling CE, Hinckley JA, Stahly EE (1950). Butadiene from ethyl alcohol - catalysis in the one-step and 2-step processes. Ind Eng Chem.

[14] Corson BB, Stahly EE, Jones HE, Bishop HD (1949). Butadiene from ethyl alcohol - study of the variables of operation. Ind Eng Chem.

[15] Jones HE, Stahly EE, Corson BB (1949). Butadiene from ethanol - reaction mechanism. J Am Chem Soc.

[16] Quattlebaum WM, Toussaint WJ, Dunn JT (1947). Deoxygenation of certain aldehydes and ketones - preparation of butadiene and styrene. J Am Chem Soc.

[17] Toussaint WJ, Dunn JT, Jackson DR (1947). Production of butadiene from alcohol. Ind Eng Chem.

[18] Murray IL, Marsh JL, Va W, Smith SP (1946). US Patent.

[19] Spence LU, Park E, Butterbaugh DJ, Kundiger DG (1948). United States Pat.

[20] Lebedev SV, Gorin YA, Khutoretzkaya SN (1935). The mechanism of the catalytic conversion of alcohols into biethylene hydrocarbons. Sint Kauch.

[21] Lebedev SV (1933). Preparation of bivinyl directly from alcohol. I. Zh Obshch Khim.

[22] Kitayama Y, Michishita A (1981). Catalytic activity of fibrous clay mineral sepilolite for butadiene formation from ethanol. J Chem Soc Chem Commun.

[23] Delacaillerie JBD, Gruver V, Fripiat JJ (1995). Modification of the surface properties of natural phyllosilicate sepiolite by secondary isomorphic substitution. J Catal.

[24] Gruver V, Sun A, Fripiat JJ (1995). Catalytic properties of aluminated sepiolite in ethanol conversion. Catal Lett.

[25] Makshina EV, Janssens W, Sels BF, Jacobs PA (2012). Catalytic study of the conversion of ethanol into 1,3-butadiene. Catal Today.

[26] Kvisle S, Aguero A, Sneeden RPA (1988). Transformation of ethanol into 1,3-butadiene over magnesium-oxide silica catalysts. Appl Catal.

[27] Ohnishi R, Akimoto T, Tanabe K (1985). Pronounced Catalytic Activity and Selectivity of MgO-SiO_2_-Na_2_O for Synthesis of Buta-1,3-diene from Ethanol. J Chem Soc Chem Commun.

[28] Kitayama Y, Satoh M, Kodama T (1996). Preparation of large surface area nickel magnesium silicate and its catalytic activity for conversion of ethanol into buta-1,3-diene. Catal Lett.

[29] Lewandowski M, Babu GS, Vezzoli M, Jones MD, Owen RE, Mattia D, Plucinski P, Mikolajska E, Ochenduszko A, Apperley DC (2014). Investigations into the conversion of ethanol to 1,3-butadiene using MgO:SiO2 supported catalysts. Catal Commun.

[30] Niiyama H, Echigoya E, Morii S (1972). Butadiene formation from ethanol over silica-magnesia catalysts. B Chem Soc Jpn.

[31] Takezawa N, Hanamaki C, Kobayashi H (1975). Mechamism of dehydrogenation of ethanol on magnesium-oxide. J Catal.

[32] Chae H-J, Kim T-W, Moon Y-K, Kim H-K, Jeong K-E, Kim C-U, Jeong S-Y (2014). Butadiene production from bioethanol and acetaldehyde over tantalum oxide-supported ordered mesoporous silica catalysts. Appl Catal B-Environ.

[33] Ezinkwo GO, Tretjakov VF, Talyshinky RM, Ilolov AM, Mutombo TA (2014). Creation of a continuous process for bio-ethanol to butadiene conversion via the use of a process initiator. Catal Commun.

[34] Jones MD, Keir CG, Di Iulio C, Robertson RAM, Williams CV, Apperley DC (2011). Investigations into the conversion of ethanol into 1,3-butadiene. Catal Sci Technol.

[35] Ordomskii VV, Sushkevich VL, Ivanova II, Ordomskiy VV, Ivanova LL (2012). Patent WO2012015340-A1.

